# The relationship between maternal attachment, maternal self-efficacy, and postpartum depressive symptoms in mothers during the early postpartum period: a cross-sectional study

**DOI:** 10.1186/s12884-026-09177-z

**Published:** 2026-04-29

**Authors:** Kadriye Ozyazici

**Affiliations:** Department of Child Development, Sivas Cumhuriyet University, Sivas, Türkiye

**Keywords:** Postpartum depression, Maternal self-efficacy, Maternal attachment, Mother-infant bonding, Early postpartum period

## Abstract

**Background:**

The postpartum period involves critical adjustments where maternal mental health and mother-infant bonding are paramount. While postpartum depression is a known risk factor, the mechanisms linking it to maternal attachment, particularly through the lens of maternal self-efficacy, require further investigation. This study aims to examine the relationship between postpartum depression, perceived maternal parenting self-efficacy, and maternal attachment, and to test the mediating role of self-efficacy.

**Methods:**

This quantitative, correlational study involved 345 mothers recruited from one family health center in Sivas, Turkey, between October 2024 and March 2025. Data were collected using a Personal Information Form, the Perceived Maternal Parenting Self-Efficacy Scale (PMPS-E), the Postpartum Depression Screening Scale (PDSS), and the Maternal Attachment Inventory (MAI). Structural equation modeling (SEM) with the WLSMV estimator was used to test the hypothesized mediation model. Participants were predominantly aged 26–30 years (37.97%), held a university degree (47.83%), were homemakers (72.17%), and reported a middle-income level (84.06%).

**Results:**

A significant negative association was also found between maternal self-efficacy and maternal attachment. Given that higher scores on both the PMPS-E and MAI indicate higher levels of self-efficacy and attachment, respectively, this inverse relationship was considered unexpected and was interpreted cautiously. No significant direct relationship was found between postpartum depressive symptoms and maternal attachment. The key finding was that perceived maternal parenting self-efficacy showed a statistically significant indirect effect in the relationship between postpartum depressive symptoms and maternal attachment.

**Conclusions:**

The findings suggest that postpartum depressive symptoms may not directly impair maternal attachment but operate indirectly by undermining mothers’ perceived parenting self-efficacy, underscoring the critical protective function of self-efficacy. Interventions aimed at enhancing maternal self-efficacy could be vital in mitigating the adverse effects of depressive symptoms on mother-infant bonding.

**Supplementary Information:**

The online version contains supplementary material available at 10.1186/s12884-026-09177-z.

## Introduction

The emotional bond between mother and baby is one of the most critical relationships in early life. It is of fundamental importance for both the baby’s psychosocial development and the mother’s mental health [1]. This bond is a dynamic process that begins during pregnancy and gains a new dimension with birth [2]. While early psychoanalytic perspectives, such as Freud’s, emphasized attachment as emerging from the mother’s fulfillment of the infant’s physiological needs [3, 4], John Bowlby [5] later developed a more comprehensive theoretical framework, conceptualizing attachment as a biologically based behavioral system. According to Bowlby, humans are biologically predisposed to form attachment relationships with primary caregivers; a caregiver who is accessible and responsive in times of need promotes a sense of stable attachment. This bond occurs through emotional signals such as smiling, crying, and sucking [6]. For secure attachment, children need to experience a warm, close, and continuous relationship with their mothers, in which the mother serves as a secure base [6, 7].

According to attachment theory, infants develop expectations about their caregivers based on the quality of care. These expectations form the basis of mental representations called “internal working models” that affect emotional functioning throughout life [8]. The bond between mother and infant is reciprocal and they influence each other [9]. People’s innate psychobiological systems motivate them to seek proximity to significant attachment figures when in need. Interactions with attachment figures who are accessible when needed and responsive to offers of proximity and support promote a stable sense of attachment [5]. However, interactions with inconsistent or unresponsive caregivers hinder the development of a secure mental foundation and make the individual psychologically more vulnerable [8]. An individual’s own early care experiences form their parenting schemas. This leads to negative self-evaluation, negative mood [10], and puts them at greater risk for postpartum depression [11]. Therefore, insecure attachment style is significantly associated with depression [11–15]. Insecure schemas can undermine relationships by operating outside of awareness and create a predisposition to depression with feelings of loss and loneliness [11]. Postpartum depression is among the common mood disorders seen in the postpartum period [16]. Mothers with high levels of postpartum depression establish less positive relationships with their children, experience more stress, and evaluate themselves as having less secure attachment [17]. Depressed mothers are more likely to have an insecure state of mind [18], and the quality of life of women who think they cannot cope with this situation is significantly affected [11].

The transition to parenthood is conceptualized as a general life stressor. During this process, parental self-efficacy, which reflects the mother’s belief in coping with caregiving responsibilities, plays a critical role. Self-efficacy is a protective psychological factor against parental dissatisfaction, stress, and depressive symptoms [19–22]. Parents’ self-efficacy beliefs reduce the risk of reactivity and depression [23–25], while increasing their beliefs in problem-solving skills, sensitivity, and responsibility [26]. Therefore, parental self-efficacy has a significant negative relationship with depression [27, 28].

According to Bandura [19, 29], self-efficacy is an individual’s belief in their ability to organize and sustain the necessary actions to achieve their goals. Parental self-efficacy is associated with sensitive and responsive caregiving [27, 30–32]. Parents’ beliefs in their abilities to raise their children in a healthy and success-promoting manner support healthy functioning for both parents and children. Negative parenting is a risk factor in the development of a range of health and behavioral problems in childhood and is a predictor of poor adult behaviors [25, 33]. Additionally, parental self-efficacy mediates children’s social-emotional development [34], aggression, exclusion, fear, anxiety, hyperactivity, and peer victimization [35], behavioral problems [36] and future criminal behavior in children [37]. Considering that parental self-efficacy is related to the quality of parent-infant interactions [38], parents’ interactions with their children play a central role in individuals’ sense of competence [39]. In the motherhood experience, parental self-efficacy positively affects mental health [27] and marital satisfaction [31]. Mothers with a history of attachment trauma and experiencing postpartum depressive symptoms are particularly at high risk for low self-efficacy [30].

**Fig. 1:**
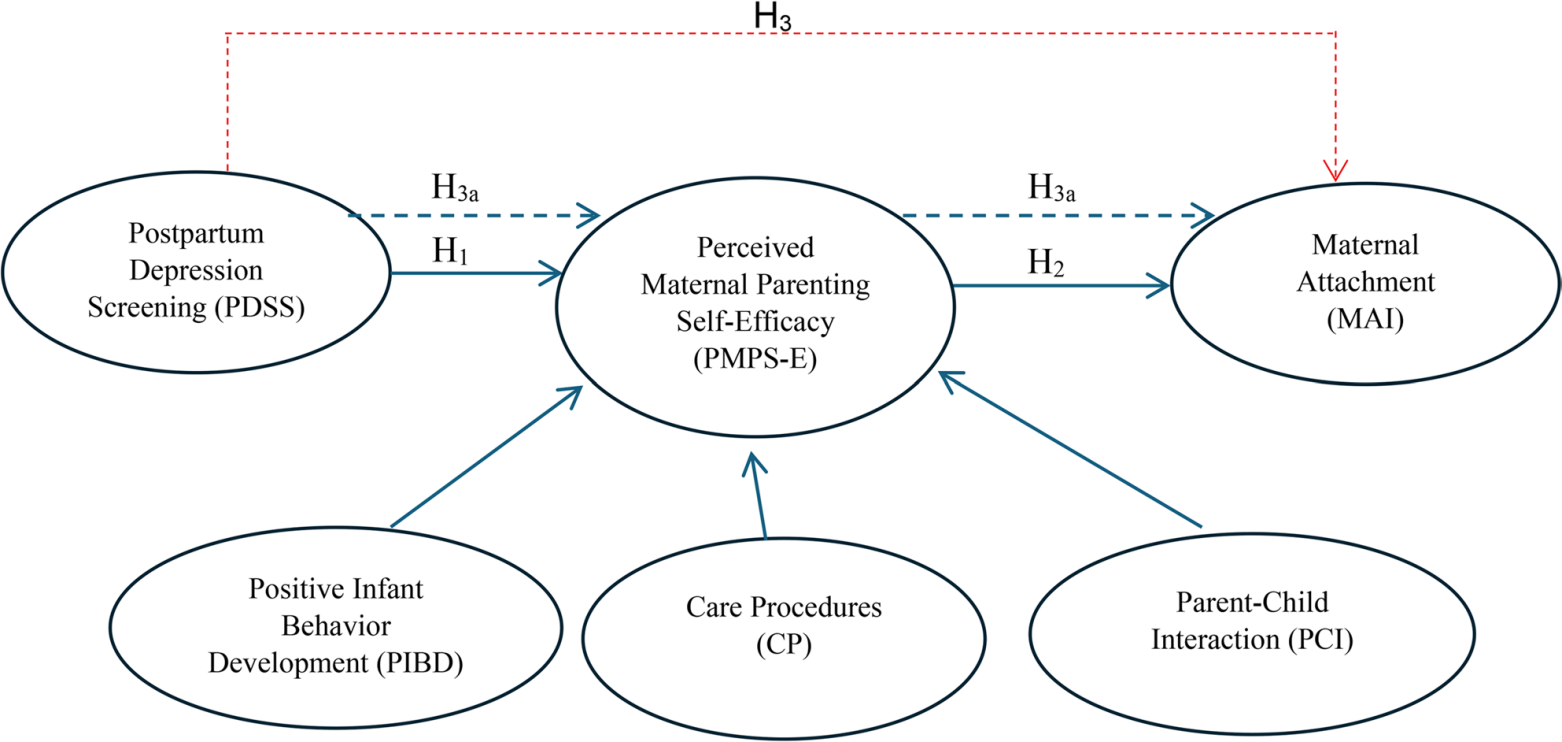
Research hypotheses

Although the associations among postpartum depressive symptoms, maternal self-efficacy, and maternal attachment have been explored separately, the mechanisms linking these variables remain underexamined. Specifically, the potential mediating role of self-efficacy in the relationship between depressive symptoms and mother-infant attachment has not been sufficiently tested. Addressing this gap, the present study aims to examine these relationships and, as illustrated in Fig. 1, test whether perceived maternal parenting self-efficacy mediates the association between postpartum depressive symptoms and maternal attachment. The hypotheses of this research are as follows:

- H₁: There is a significant relationship between Postpartum Depression and Maternal Attachment.
- H₂: There is a significant relationship between Perceived Maternal Parenting Self-Efficacy and Maternal Attachment.
- H₃: Perceived Maternal Parenting Self-Efficacy has a mediating role in the relationship between Postpartum Depression Screening and Maternal Attachment.

## Methods

This study is a quantitative and correlational research examining the relationships between postpartum depression, maternal parenting self-efficacy, and maternal attachment. This model is a research design in which the researcher examines the relationship between two or more variables without any intervention on the variables, aiming to make predictions based on these relationships [40, 41]. This design allows for statistical testing of the interaction of variables in their natural environment and describing predictive relationships. Accordingly, in the research, postpartum depression (PDSS) was positioned as the predictor variable, maternal attachment (MAI) as the outcome variable; and maternal parenting self-efficacy (PMPS-E) was considered as a mediator variable in this relationship.

### Participants

The study sample consisted of 345 mothers from one family health center in Sivas province, Turkey, between October 2024 and March 2025. For the purposes of this study, the postpartum period was defined as the first month after birth, consistent with the validation periods of the scales used [42–44]. Eligible participants were mothers of healthy, singleton infants aged 0–1 months, aged 18 years or older, able to read and write Turkish, and without communication barriers. Mothers were included regardless of parity (i.e., both primiparous and multiparous mothers were eligible). Exclusion criteria were age under 18 years, inability to read and write Turkish, lack of willingness to participate, and withdrawal from the study at any stage.

Table 1 shows the demographic characteristics of the mothers who participated in the study. The research sample consists of 345 participants. When examining the age distribution of participants, the highest percentage was in the 26–30 age group with 37.97% (*n* = 131). This group was followed by the 31–35 age group with 26.96% (*n* = 93) and the 18–25 age group with 21.45% (*n* = 74), respectively. The 36–40 age group represented 10.43% (*n* = 36), while those over 40 years of age accounted for 3.19% (*n* = 11). According to education level, nearly half of the mothers were university graduates (47.83%, *n* = 165). Middle school graduates constituted 24.93% (*n* = 86), high school graduates 23.77% (*n* = 82), and primary school graduates 3.48% (*n* = 12). In terms of employment status, the majority of participating mothers were housewives (72.17%, *n* = 249), while 27.83% (*n* = 96) worked in income‑generating jobs. Regarding household income level, a large proportion of participants identified themselves as middle‑income level (84.06%, *n* = 290). Low income level was reported by 10.14% (*n* = 35), and high income level by 5.80% (*n* = 20).

**Table 1:** Demographic characteristics of the study sample (*n* = 345): distribution by age, education level, employment status, and household income level

Demographic variables	*N*	%
Age		
18–25 age	74	21.45
26–30 age	131	37.97
31–35 age	93	26.96
36–40 age	36	10.43
40 and over	11	3.19
Education level		
Primary School	12	3.48
Middle School	86	24.93
High School	82	23.77
Undergraduate	165	47.83
Employment status		
Working in an income-generating job	96	27.83
Housewife	249	72.17
Household income level		
Low	35	10.14
Medium	290	84.06
High	20	5.8

### Data collection tools

In this study, a personal information form was administered to obtain demographic data from the participants, along with the “Perceived Maternal Parenting Self-Efficacy Scale”, the “Postpartum Depression Screening Scale” and the “Maternal Attachment Inventory”. Brief information about these scales and evidence of their validity and reliability within the study group are provided.

#### Demographic ınformation form

The Demographic Information Form consisted of 4 questions developed by the researcher based on a review of relevant literature [42–44]. The form collected data on participants’ age, educational level, employment status, household income level.

#### Perceived Maternal Parenting Self-Efficacy Questionnaire (PMPS-E)

The scale was developed by Barnes and Adamson-Macedo [45] to measure the perceived parenting self-efficacy of mothers with babies. The Perceived Maternal Parenting Self-Efficacy Scale consists of 20 items and 4 sub-dimensions. The scale is a Likert-type scale composed of positive items, with each item being evaluated between strongly disagree (1) and strongly agree (4). As the score obtained from the scale increases, the mothers’ self-efficacy level increases [45]. In the original form of the scale, the Cronbach’s alpha coefficient was determined to be 0.91. The sub-dimension Cronbach’s alpha coefficients were found to be 0.74 for “1st sub-dimension”, 0.89 for “2nd sub-dimension”, 0.74 for “3rd sub-dimension”, and 0.72 for “4th sub-dimension”. The scale, which was adapted to Turkish by Verici Kılıç and Kavlak, consists of 18 items and three sub-dimensions. These sub-dimensions are “mother-infant interaction” comprising items 1,2,4,5,6,7,8,13,18; “care procedures” comprising items 3,14,15,16,17; and “belief in developing positive infant behaviors” comprising items 9,10,11,12. Each item on the scale is evaluated between strongly disagree (1) and strongly agree (4). As the score obtained from the scale increases, the mothers’ self-efficacy level increases. A general score is obtained from the sum of all items. Content Validity Index scores were found to be between 0.90 and 1.00. With Exploratory Factor Analysis (EFA), the “Kaiser-Meyer-Olkin (KMO) value was determined to be 0.932 and the Barlett’s Test of Sphericity was p < 0.001. As a result of the factor analysis, it was determined that the scale consists of three sub-dimensions and explains 65.815% of the total variance. As a result of the Confirmatory Factor Analysis, it was confirmed that the three-factor structure of the scale was valid, and the X²/df ratio was 2.608, CFI value was 0.930, NFI value was 0.892, GFI value was 0.876, and RMSEA value was 0.080 [42]. In this study, the goodness-of-fit indices of the CFA analysis for the three-dimensional Perceived Maternal Parenting Self-Efficacy Scale are presented in Supplementary Table S1. As shown in Supplementary Table S1, the scale demonstrates an acceptable level of fit in the sample to which it was applied (χ² = 495.44; df = 125; CFI = 0.90; TLI = 0.87; RMSEA = 0.09). These values indicate that the structural validity of the scale is adequate and that it demonstrates strong validity in representing the construct it measures.

#### Postpartum Depression Screening Scale (PDSS)

The PDSS is a 35-item self-report scale with 7 subscales, each containing 5 items, rated on a 5-point Likert scale. Each item describes how the mother has been feeling since the birth of her baby. When completing the scale, women are asked to rate each item on a scale from strongly disagree (1) to strongly agree (5), based on their feelings over the past two weeks. The total score obtainable from the scale ranges from 35 to 175. The alpha internal consistency reliability for each dimension is as follows: Sleep/Eating Disturbances, 0.83; Anxiety/Insecurity, 0.83; Emotional Lability, 0.89; Cognitive Impairment, 0.91; Loss of Self, 0.94; Guilt/Shame, 0.89; Contemplating Self-harm, 0.93. In a study where clinical interviews using DSM-IV were taken as the standard, when the PDSS cut-off point was set at 80 for determining major depression, the sensitivity was reported as 0.94, specificity as 0.98, positive predictive value as 0.90, and negative predictive value as 0.99. For determining minor or major depression, when the PDSS cut-off point was set at 60, the sensitivity was reported as 0.91, specificity as 0.72, positive predictive value as 0.59, and negative predictive value as 0.95 [46]. The original PDSS was adapted to Turkish by Karaçam and Kitiş [43] after conducting validity and reliability studies. The construct validity of the adapted scale was determined using confirmatory factor analysis and item response theory techniques. The reliability of the scale was determined by calculating item analysis, alpha internal consistency reliability, and dimension-level reliability. In Turkish, the PDSS’s Cronbach’s Alpha internal consistency coefficient was found to be 0.94, test-retest reliability was *r*=0.86, and the equivalence coefficient for the split-half test was *r*=0.91. Exploratory factor analysis revealed that the scale consists of 6 factors with eigenvalues above 1, which together account for 54.69% of the total variance. In the Turkish version of PDSS, all items were found to belong to a factor with positive loadings between 0.34 and 0.77. The item-total test correlation coefficients of the Turkish PDSS were calculated to be between 0.35 and 0.68, and the item-total subscale correlation coefficients were between 0.31 and 0.71, indicating sufficient discriminatory power [43]. In this study, the goodness-of-fit indices from the CFA analysis are presented in Supplementary Table S2. As shown in Supplementary Table S2, the Postpartum Depression Screening Scale demonstrates a partially acceptable level of fit in the study sample (χ² = 1138.29; df = 438; CFI = 0.88; TLI = 0.85; RMSEA = 0.09). Although the CFI and TLI values approached the 0.90 threshold recommended in the literature, they did not fully meet this cutoff. The RMSEA value being in the range of 0.08–0.10 indicates that the model has a poor fit. These findings suggest that the structural validity of the scale is generally acceptable, albeit limited in the applied sample, but the fit could be improved with model refinement. The Cronbach’s Alpha reliability coefficient was calculated for internal consistency reliability and was found to be 0.95. Because the reliability coefficient exceeded 0.70, the scale yielded reliable results in this sample.

#### Maternal Attachment Inventory (MAI)

The Maternal Attachment Inventory was developed by Mary E. Muller in [47] to measure maternal attachment with love. Muller initially applied the scale to 196 mothers with babies between 30 and 40 days postpartum. The reliability coefficient of MAI was found to be Cronbach’s alpha 0.85. The correlation between MAI and “How I Feel About My Baby Now” (HIFBN) was determined as *r* = 0.45, *p* < 0.001, and with the Maternal Separation Anxiety Scale (MSAS) as *r* = 0.46, *p* < 0.001 [47, 48]. In the second stage, to examine whether MAI could be used after the postpartum period, Muller applied it to a group (*n* = 62) of these 196 mothers at the fourth month after birth, and to another group (*n* = 86) at the eighth month after birth. Cronbach’s alpha was found to be 0.76 at the fourth month postpartum and 0.85 at the eighth month. Although the Maternal Attachment Inventory was prepared as a 31-item scale, five items were removed from the scale as they were found to have low psychometric correlation. Consequently, MAI was accepted as 26 items [47, 48]. Each item contains direct statements and is scored as always (4) and never (1). A general score is obtained from the sum of all items. A high score indicates high maternal attachment. The lowest possible score from the scale is 26, and the highest is 104 [47–49]. After conducting language validity studies for the Maternal Attachment Inventory, analysis based on the scores given by eight experts for the content validity of the 26 items (Kendall’s W = 0.274 (*p* = 0.001 < 0.01)) revealed that the experts reached a consensus on the content of the items. The Cronbach’s Alpha internal consistency reliability of the Maternal Attachment Inventory was determined to be 0.77 for mothers with 1-month-old babies and 0.82 for mothers with 4-month-old babies. A high score from the scale indicates high maternal attachment. The correlation coefficient between the first and second applications for test reliability was determined to be 0.596, and a statistically significant relationship was found at the α = 0.001 level (*r* = 0.596, *p* < 0.001). The reliability coefficient of MAI was found to be Cronbach’s alpha 0.85 [44]. In this study, the goodness-of-fit indices for the CFA analysis of the Maternal Attachment Inventory (MAI) are presented in Supplementary Table S3. As shown in Supplementary Table S3, the MAI demonstrates good fit in the study sample (χ² = 66.886; df = 19; CFI = 0.94; TLI = 0.91; RMSEA = 0.08). CFI and TLI values being above 0.90 indicate that the model presents a strong fit; despite the RMSEA value being 0.08, it remains within the limits accepted in the literature here, supporting that the scale has a valid structural model in this sample. These findings demonstrate that the Maternal Attachment Inventory is a reliable and valid measurement tool that serves its purpose in the relevant sample. The Cronbach’s Alpha reliability coefficient calculated for the internal consistency of the scale was 0.92. Given that the reliability coefficient exceeded 0.70, the scale yielded reliable results in this sample.

### Data collection and data screening

Data were collected via face‑to‑face interviews at the participating family health centers. The researcher explained the purpose of the study, assured confidentiality, and obtained written informed consent from each participant. Mothers completed the questionnaire set independently in a quiet room; the researcher was available to answer questions. The administration took approximately 20–25 min per participant.

Working with large samples is important in multivariate statistical analyses [50, 51]. Although various criteria exist in the literature for determining sample size, Kline [50] specifically recommends conducting statistical power analysis. Accordingly, in the power analysis performed in this research, when the effect size was set at 0.30, significance level (α) at 0.05, and test power (1 − β) at 0.95, the recommended minimum sample size was calculated as 136.

After completing outlier and missing data procedures, analyses continued with 345 participants. This value is well above the minimum sample size of 136 recommended by the power analysis. Therefore, it was concluded that the sample size of the research was sufficient and powerful for multivariate statistical analyses.

The data obtained from the study group were first examined in terms of missing data, univariate and multivariate outliers, univariate and multivariate normality, linearity, and multicollinearity. As a result of the data examination, it was determined that there were no missing data. Within the scope of outlier analysis, 18 outliers exceeding the criterion of |3.29 [52] according to z standard scores were identified and excluded from the analysis. Following the absence of missing data and the removal of outliers, analyses were conducted with 345 participants.

In the normality examination, skewness and kurtosis coefficients were evaluated; as suggested by Tabachnick and Fidell [52], values within the limits of |3.0| were accepted as meeting the normal distribution assumption. In this research, it was observed that all skewness and kurtosis values of the variables were within this range (Table 2).

**Table 2:** Descriptive statistics for the dataset used to test the hypotheses

Variables	X	SS	Skewness	Kurtosis	Tolerance	VIF
MAI	11.39	2.78	2.11	6.13	13.98	0.072
PMPS-E	64.11	6.82	-0.44	-0.81	15.92	0.063
PDSS	56.03	19.9	0.83	-0.03	7.23	0.138

To assess the issue of multicollinearity, correlation coefficients were examined, and it was concluded that there was no multicollinearity problem since the coefficients were below 0.90 [50]. Additionally, Tolerance (> 0.10) and Variance Inflation Factor (VIF < 10) values were evaluated; Tolerance values above 0.10 and VIF values below 10 for all variables indicated that there was no collinearity problem (Table 2). When examining the Tolerance and VIF values for DSD, it was observed that Tolerance values were below 0.10. However, since the VIF value was below 10, it showed that there was no collinearity problem. Furthermore, the correlation coefficients between variables in Table 3 demonstrate that linearity was achieved and there was no connection problem.

**Table 3:** Correlation among the constructs

	MAI	PDSS	PMPS-E
MAI			
PMPS-E	-0.19	1	
PDSS	-0.04	-0.38	1

## Results

### Measurement model results: reliability and validity

Confirmatory factor analysis (CFA) was applied to test the structural validity of the measurement model used in the research. The fit indices calculated for the obtained measurement model are χ2 = 3894.394; df = 2268; *p* < 0.001; χ2/df = 1.71; TLI = 0.75; CFI = 0.76; RMSEA = 0.04[0.043–0.048]; SRMR = 0.10. Item‑level analysis was retained to preserve the full psychometric information of the scales, despite the complexity of the measurement model. While some scale‑specific modifications (e.g., residual correlations for the Maternal Attachment Inventory, as detailed in Supplementary Table S3) were implemented based on theoretical and empirical considerations, these did not substantially improve the overall model fit. The RMSEA value being below 0.05 indicates that the model demonstrated acceptable fit in terms of absolute indices (RMSEA), although the comparative fit indices (CFI and TLI) remained below recommended thresholds and should be interpreted with caution [50, 53]. However, the CFI (0.76) and TLI (0.75) values remain below the 0.90 threshold accepted in the literature. This indicates that the model has a limited fit in terms of comparative fit indices. Additionally, the SRMR value being at the level of 0.10 (above 0.08) shows that the residual covariances in the model are relatively high.

Despite testing various alternatives within the scope of model improvement attempts, such as removing items with low factor loadings and examining modification indices, no significant improvement was observed in the fit indices. This situation may likely stem from the original factor structure of the scale or sample-specific variability. Particularly, multidimensional emotional and behavioral constructs can yield low values in comparative fit indices in cross-cultural applications or comprehensive measurement models [54, 55].

However, the low RMSEA and the medium-high level of factor loadings for many items do not indicate that the model should be completely rejected [56]. RMSEA is especially a powerful index that evaluates the overall error level of the model, and values below 0.05 indicate that the model operates with an acceptable level of error. In other words, although the model has limited fit in comparative fit indices, it exhibits a good fit in terms of absolute fit. To strengthen the validity evidence, convergent validity and discriminant validity analyses were also conducted in the measurement model. As basic criteria for convergent validity, factor loadings exceeding 0.40 [57], average variance extracted (AVE) values exceeding 0.50 [58, 59], and composite reliability (CR) coefficients above 0.70 [60] were considered.

For convergent validity, factor loadings of at least 0.40 [57], average variance extracted (AVE) values above 0.50 [58, 59], and composite reliability (CR) coefficients exceeding 0.70 [60] were adopted as basic criteria. These criteria are considered sufficient to establish convergent validity according to both classical measurement theory and modern structural equation modeling literature [50, 61]. As presented in Supplementary Table S4, the factor loadings from the analysis ranged between 0.35 and 0.90 and were significant. For AVE values, one dimension was found to be below 0.50. Although this indicates limited convergent validity, the composite reliability values exceeded the 0.70 threshold, suggesting adequate internal consistency [62]. The low AVE for the MAI and a small number of items with factor loadings below 0.40 are acknowledged as limitations of the measurement model. Additionally, discriminant validity was calculated and interpreted using the √AVE method and is presented in Table 4.

**Table 4:** Discriminant validity and correlations among latent constructs

	PCI	CP	PIBD	MAI	PDSS
PCI	0.61				
CP	0.72	**0.69**			
PIBD	0.67	0.69	**0.83**		
MAI	-0.32	-0.22	-0.15	**0.64**	
PDSS	-0.31	-0.37	-0.40	0.016	**0.64**

p* < 0.001 for all correlations, Bold diagonal values represent the square roots of AVE (Fornell–Larcker criterion)

It has been suggested that the value should be below 0.85 for discriminant validity [50]. These values indicate that the scales have adequate discriminant validity.

In this context, the limitation in the comparative fit indices of the measurement model has been discussed in the limitations section of the article; however, the model continued to be used in the analyses, taking into account the theoretical framework and content validity of the items.

### Hypothesis testing and results

In this section, hypotheses H1, H2, and H3 were tested to examine the regression between the Perceived Maternal Self-Efficacy (PMPS-E) variable’s subdimensions Parent-Child Interaction (PCI), Care Procedures (CP), and Positive Infant Behavior Development (PIBD) and the variables of Postpartum Depression Screening Scale (PDSS) and Maternal Attachment Inventory (MAI). The relevant coefficients are provided in Table 5.

**Table 5:** Hypotheses results

Hypothesis	Path	β	t-value	*p*-value	Status
H1	PMPS-E→ MAI	-0.347	-6.030	< 0.001	Accepted
H2	PDSS → PMPS-E	-0.463	-11.879	< 0.001	Accepted
H3	PDSS → MAI	-0.082	-1.226	0.220	Rejected

𝜒^2^ = 3894.394, sd = 2268, RMSEA = 0.046 [0.043–0.048], CFI = 0.76, TLI = 0.75, SRMR = 0.10

After the measurement model was validated, the fit indices of the structural equation model established to test the relationships between latent variables are as follows: χ²(2268) = 3894.394, RMSEA = 0.046, 90% CI [0.043–0.048], CFI = 0.76, TLI = 0.75, SRMR = 0.10. The RMSEA value being below 0.05 and the entire confidence interval being less than 0.05 indicates that the model has good absolute fit. Additionally, the χ²/df ratio of approximately 1.71 (3894.394 / 2268 ≈ 1.71) supports the overall fit of the model as it falls below the recommended threshold of 2.00 in the literature. Conversely, the CFI (0.76) and TLI (0.75) values were observed to be below the threshold value of 0.90. This indicates that the model has limited fit in terms of comparative fit indices. The SRMR value of 0.10 suggests that residual covariances are relatively high, indicating that some item-level errors are not adequately explained by the model. Various studies have emphasized that in models with multiple items (PDSS 35 items), multiple factors (postpartum depression with six factors), and categorical items (Perceived Maternal Self-Efficacy), CFI and TLI can systematically yield lower values [55, 63]. Taken together, these findings suggest that the structural model exhibits acceptable absolute fit (based on RMSEA and χ²/df), yet the comparative fit indices (CFI and TLI) indicate limited model fit. Therefore, the model was evaluated as a partially acceptable structural model based on absolute fit and theoretical framework, and this limitation was taken into account when interpreting the findings.

The paths and standardized coefficients (STDYX) tested with the structural model were examined. According to these coefficients, as the level of postpartum depressive symptoms increases, perceived parental self-efficacy changes significantly and negatively (β = -0.463, *p* < 0.001). In other words, if postpartum depressive symptoms is high, the parent’s perception of self-efficacy is significantly low. Similarly, perceived parental self-efficacy showed a significant negative association with maternal attachment (β = − 0.347, *p* < 0.001). According to the established scoring of the PMPS‑E (where higher scores reflect greater self‑efficacy) and the MAI (where higher scores reflect stronger attachment), this result indicates that lower self‑efficacy was associated with higher attachment scores in this sample. This means that maternal attachment is higher in parents with low self-efficacy. In contrast, the direct effect of postpartum depressive symptoms on maternal attachment was not found to be statistically significant (β = -0.082, *p* = 0.220).

When examining the R² values, the explained variance ratios obtained for latent variables are as follows: R² = 0.214, S.E. = 0.036, *p* < 0.001 for PMPS-E, and R² = 0.101, S.E. = 0.032, *p* = 0.001 for MBI. These values indicate that postpartum depressive symptoms explains approximately 21.4% of perceived maternal self-efficacy. That is, although the PDSS level is only a single predictor in the model, it explains a significant portion of the variance in PMPS-E. The R² value obtained for maternal attachment indicates that 10.1% of the variance in MAI is explained by PMPS-E. According to these results, it is suggested that other psychosocial variables that may accompany the attachment process should also be included in the model for maternal attachment.

Hayes’ bootstrap method was used for the mediation test (5000 bootstrap samples, 95% bias-corrected). The indirect effects of the Maternal Self-Efficacy (PMPS-E) variable with Maternal Attachment (MAI) and Postpartum Depression Screening Scale (PDSS) variables are given in Table 6. The 95% confidence interval was used as a basis to determine whether there was mediation, and the mediation relationship was considered significant because the confidence interval did not include 0 [64].

**Table 6:** Mediation analysis

		Direct effect			Indirect Effect			
Hypothesis	Path	β	t-value	*p*-value	Estimate	CI(Lower)	CI(Upper)	Status
H_3a_	PDSS → PMPS-E → MAI	-0.082	-1.226	0.220	0.161	0.109	0.225	Full Mediation

According to the mediation test results, the mediation effect between the Postpartum Depression Screening Scale (PDSS) variable and Maternal Attachment (MBI) is significant (β = 0.161, CI ranged from 0.109 to 0.225). The confidence intervals not including zero supports hypothesis H3a.

After testing the existence of the mediation effect, the significance of the direct effect of the (PDSS) variable on Maternal Attachment (MAI) was examined to determine the type of mediation (full or partial). Accordingly, the direct effect between the two variables (β = − 0.082, *p* = 0.220) is not significant. Therefore, it was determined that the type of mediation is full mediation.

## Discussion

The findings of our study suggested that as the level of postpartum depressive symptoms increased, mothers’ perceived parental self-efficacy showed a significant negative trend. This finding aligns with patterns observed in several previous studies [65–69]. Previous research indicates that this relationship could be reciprocal over time. For example, one longitudinal study revealed that higher levels of depression predicted lower parental efficacy, while lower efficacy predicted higher depression in subsequent periods [70]. Some research results indicate that low self-efficacy may be associated with an increased likelihood of reporting depressive symptoms [18]. It is proposed that the negative cognitive processes and behaviors of depressed mothers can contribute to decreased parental self-efficacy [71]. Postpartum depressive symptoms has been linked to negative effects on perceived competence and satisfaction with the maternal role shortly after birth [72]. On the other hand, high self-efficacy is thought to enable mothers to cope more resiliently with postpartum challenges [73] and to use their internal resources and coping strategies more effectively [74]. Consistent with Bandura’s [19] theoretical framework, self-efficacy is considered one of the factors closely related to an individual’s expectations, resilience, and vulnerability [75]. Therefore, increases in self-efficacy are often correlated with a decrease in depressive symptoms [69].

During the postpartum period, rapid changes in hormones can increase vulnerability to mood disturbances and the risk of depression. Consequently, postpartum depressive symptoms might lead to feelings of helplessness, inadequacy, difficulty concentrating, and intense anxiety about infant care [67]. These negative emotions could potentially impact the mother’s self-confidence and reduce her belief in her competence in essential skills, particularly breastfeeding [76]. In this process, mothers with high mental health literacy may gain more self-efficacy in managing stress factors through knowledge acquisition and developing positive attitudes. This could allow them to exhibit more effective health behaviors [77]. However, in the literature, there are also findings suggesting that the general trend in the relationship between postpartum depressive symptoms and parental self-efficacy could be influenced by cultural context. A study conducted among undocumented migrant mothers shows the co-occurrence of high levels of postpartum depressive symptoms with high maternal self-efficacy. This revealed that in this group, depression may not show the expected direct negative effect on self-efficacy [78]. This result provides a clue that perceptions of depression and caregiving confidence may differ culturally.

Another finding obtained in the study showed a significant and negative relationship between mothers’ perceived maternal self-efficacy and maternal attachment. According to the scoring of the Perceived Maternal Parenting Self-Efficacy Scale (PMPS-E), where higher scores indicate greater self-efficacy [43], and the Maternal Attachment Inventory (MAI), where higher scores indicate stronger attachment [44], this negative coefficient (β = − 0.347) indicates that lower self-efficacy was associated with higher attachment scores in this sample. This finding contrasts with the more commonly reported view in the existing literature, which generally supports a positive relationship between higher maternal self-efficacy and stronger or higher quality mother-infant attachment [79–82]. Similarly, insecure or disrupted attachment has been associated with low self-efficacy in some studies [83, 84]. Previous studies indicate a relationship between strong attachment and subsequent high self-efficacy [80], suggest that intervention studies’ increase in self-efficacy could mediate positive developments in attachment [85] and show that health education interventions can improve both variables simultaneously [86]. Theoretically, mothers with a strong belief in their parenting tasks might be expected to be more responsive to their babies’ needs and potentially establish stronger emotional bonds [82]. Maternal self-efficacy is viewed as one of the key components in a mother’s adaptation to motherhood and can affect not only the mother’s psychological well-being but also the child’s psychosocial development [87]. Maternal self-efficacy, the cognitive belief mothers have in their ability to perform newborn care tasks, is regarded as one of the most important components for a smooth transition to motherhood [88]. However, not all studies in literature provide consistent results. Bridges, Groner, and Mehta [2021] reported in their study that the correlation between self-efficacy and attachment was statistically significant but very weak. Some studies found no significant correlation between general self-efficacy scores and maternal attachment [81, 89–91]. This inconsistency suggests that the relationship between the two variables may be influenced by mediating variables. These results may be attributed to factors such as cultural differences, measurement tools used, or sample characteristics.

Another key finding of the study is that no significant direct relationship was detected between postpartum depressive symptoms and maternal attachment in our analysis. This finding suggests that postpartum depressive symptoms might exert an indirect rather than direct effect on attachment processes in our sample. Indeed, current models in the field of attachment suggest that parental depression can indirectly shape the bond with the baby, primarily by affecting mentalization ability and parental self-efficacy [68, 85, 92]. These results are also consistent with research showing that not all attachment difficulties stem from depression; other factors such as trauma, insecure adult attachment patterns, or cognitive distortions may also play a role [93–95].

When examining the literature, it is observed that findings on this topic are inconsistent. On one hand, there are studies showing no significant relationship between postpartum depressive symptoms and infant care quality [20, 96]. On the other hand, many studies have reported that postpartum depressive symptoms has a direct negative effect on the mother-infant bond, with higher depressive symptoms being associated with decreased sensitivity, less warm interaction, and ultimately more attachment difficulties [68, 97, 98]. It is emphasized that depression can reduce the quality of care by affecting how the mother interacts with and responds to her baby [99], negatively impacts maternal functionality [100], and may even increase the risk of neglect in severe cases [101]. This apparent contradiction indicates that the nature of the relationship is likely complex and may depend on mediating variables rather than being simple and linear. Various models suggest that the effect of depression may emerge through mediating factors such as insecure adult attachment, low self-efficacy, or decreased mentalization [12, 93, 102, 103]. Negative cognitive processes and behaviors common in depressed mothers might primarily weaken parental self-efficacy [71] rather than directly disrupting attachment, which could indirectly affect the quality of attachment. Consequently, the absence of a direct significant relationship in our study points to the possibility that this relationship is not universally direct and may be strongly influenced by contextual, methodological, and individual factors.

One of the key findings of this study is that parenting self-efficacy may have a mediating role in the relationship between postpartum depressive symptoms and maternal attachment. Our findings suggest that the effect of postpartum depressive symptoms on attachment may occur through an indirect pathway, via reductions in mothers’ perceived parenting self-efficacy, rather than solely through a direct effect. This finding is generally consistent with previous research suggesting that self-efficacy can function as a mediator in this relationship. For instance, Qi et al. [68] reported that parenting self-efficacy, alongside mentalizing, was associated with the link between postpartum depression and mother-infant bonding in a large Chinese sample. Similarly, Dlamini et al. [66] found that maternal self-efficacy played a mediating role in the relationship between postpartum depression and maternal role competence in a South African sample. Oh and Ahn [104] also identified parenting competence, together with marital satisfaction, as part of a serial mediation model in Korean mothers. A growing body of research indicates that postpartum depressive symptoms are associated with lower levels of parenting self-efficacy [18, 78, 105], and that reduced self-efficacy is, in turn, related to less optimal mother-infant attachment outcomes [66, 68, 85]. Accordingly, parenting self-efficacy has been considered a potential mediating mechanism in the relationship between postpartum depressive symptoms and parent-infant attachment [104]. Consistent with the broader literature, higher levels of parenting self-efficacy are generally associated with more positive mother-infant relationships and more secure attachment patterns [78, 106, 107]. Conversely, lower maternal self-efficacy has been linked to an increased risk of postpartum depressive symptoms, which may negatively influence maternal attachment [68, 108]. These findings suggest a potentially reciprocal or cyclical relationship in which self-efficacy plays an important role. In addition, some studies suggest that postpartum depressive symptoms may act as a mediator between other risk factors, such as attachment trauma, and parenting self-efficacy. For example, mothers with a history of attachment trauma may be more vulnerable to postpartum depressive symptoms, which could in turn be associated with lower self-efficacy [92]. However, it is also important to note that other variables such as mentalization [68] or social support [92] may play a mediating role depending on the sample and context. Taken together, these findings highlight the importance of contextual and cultural factors, suggesting that the mediating role of self-efficacy may vary across different populations. In the Turkish context, where this study was conducted, parenting self-efficacy appears to be an important explanatory factor, potentially reflecting the cultural emphasis on maternal competence.

### Clinical and research implications

The cross‑sectional design of this study prevents causal inferences; therefore, the observed indirect effect should be interpreted as a statistical association that is consistent with a mediation hypothesis rather than as evidence of a causal pathway. Nevertheless, the findings suggest that maternal self‑efficacy could be a valuable target for future research aiming to understand how depressive symptoms may influence mother‑infant attachment. From a clinical perspective, interventions designed to enhance maternal self‑efficacy such as skill‑building sessions, positive reinforcement, and peer support groups might be considered as promising strategies to explore in longitudinal and experimental studies, though the present data cannot confirm their effectiveness in mitigating attachment difficulties.

### Strengths of the study

This study has several methodological strengths that enhance its contribution to literature. First, the use of structural equation modeling (SEM) with the WLSMV estimator allowed for the simultaneous testing of the measurement model and the hypothesized mediation pathways, providing a more rigorous analysis than bivariate or regression‑based approaches. Second, the investigation of the mediating role of maternal self‑efficacy in the relationship between postpartum depressive symptoms and maternal attachment addresses an important gap in the existing literature.

### Limitations of the study

This study has several limitations that should be considered when interpreting the findings. First, the cross-sectional design prevents causal inferences about the relationships between postpartum depressive symptoms, self-efficacy, and maternal attachment. Second, the sample consisted predominantly of married, middle-income, and highly educated mothers from a specific region in Turkey, which may limit the generalizability of the results to other populations, such as single mothers, those with lower socioeconomic status, or different cultural contexts. Third, all data were based on self-report measures, which are susceptible to social desirability and recall bias. Fourth, the use of complex scales (PDSS, PMPS-E, MAI) with multiple sub-dimensions within a single structural equation model increased statistical complexity and required a substantial sample size. Finally, while appropriate estimation methods were employed (e.g., WLSMV for non-normal ordinal data), the model’s fit may have been influenced by high residual correlations among some scale items, suggesting potential shared variance beyond the theoretical constructs. These methodological considerations should be acknowledged when interpreting the model’s results. Additionally, the study did not collect data on potentially relevant variables such as whether the pregnancy was planned, the presence of social support systems, breastfeeding status, or previous parenting experience. These factors may influence maternal self-efficacy and attachment and should be addressed in future research. The complexity of the measurement model, which retained all items from three multi‑dimensional scales, contributed to suboptimal comparative fit indices. Alternative approaches such as item parceling or the use of scale‑level composites could be considered in future research to achieve more parsimonious model fit.

## Supplementary Information

Supplementary Material 1.

## Data Availability

The datasets generated and/or analyzed during the current study are not publicly available but are available from the corresponding author upon reasonable request.
