# Supplementary material for: The relationship between maternal attachment, maternal self-efficacy, and postpartum depressive symptoms in mothers during the early postpartum period: a cross-sectional study
Source: BMC Pregnancy Childbirth. 2026 Apr 29;26:651. doi: 10.1186/s12884-026-09177-z (PMC13270851; doi:10.1186/s12884-026-09177-z)
Supplement: Supplementary file 1 — Supplementary Material 1. [file 12884_2026_9177_MOESM1_ESM.docx]

**Supplementary Table**

**Supplementary Table S1**

Goodness-of-Fit Values for the Perceived Maternal Parenting Self-Efficacy Scale

| **Goodness-of-Fit Indices** | **Post-Modification Values** | **Acceptable Criteria** |  |
| --- | --- | --- | --- |
| S-B^a^ 𝜒2 | 495.440^c^ |  |  |
| sd | 125 |  |  |
| RMSEA [%90 C.I.] | .09[.08; 0.10] | ≤ .10 (Kline, 2015) |  |
| CFI | .90 | ≥ .90 (Brown, 2015) |  |
| TLI | .87 | ≥ .90 (Brown, 2015) |  |
| SRMR | .05 | ≤ .10 (Kline, 2015) |  |
| a Satorra-Bentler scaled chi-square. ^b^p=.000. ^c^p=.000. | | | |

**Supplementary Table S2**

Goodness-of-Fit Values for the Postpartum Depression Screening Scale

| **Goodness-of-Fit Indices** | **Values** | **Acceptable Criteria** |
| --- | --- | --- |
| S-B^a^ 𝜒^2^ | 1138.290^b^ |  |
| sd | 438 |  |
| RMSEA [%90 C.I.] | .09[.08; .10] | ≤ .10 (Kline, 2015) |
| CFI | .88 | ≥ .90 (Brown, 2015) |
| TLI | .85 | ≥ .90 (Brown, 2015) |
| SRMR | .05 | ≤ .10 (Kline, 2015) |
| a Satorra-Bentler scaled chi-square. ^b^p=.000 | | |

**Supplementary Table S3**

Goodness-of-Fit Values for the Maternal Attachment Inventory

| **Goodness-of-Fit Indices** | **Pre-Modification Values** | **Post-Modification Values** | **Acceptable Criteria** |
| --- | --- | --- | --- |
| S-B^a^ 𝜒2 | 30.612^b^ | 66.886^c^ |  |
| sd | 8 | 19 |  |
| RMSEA [%90 C.I.] | .08[.05; .12] | .08[.06; 0.10] | ≤ .10 (Kline, 2015) |
| CFI | .96 | .94 | ≥ .90 (Brown, 2015) |
| TLI | .92 | .91 | ≥ .90 (Brown, 2015) |
| SRMR | .04 | .05 | ≤ .10 (Kline, 2015) |
| a Satorra-Bentler scaled chi-square. ^b^p=.000. ^c^p=.000. | | | |

**Supplementary Table S4**

Confirmatory Factor Analysis (CFA) Results

| Construct and Items | Factor Loading | AVE | Alpha | CR |
| --- | --- | --- | --- | --- |
| Maternal Attachment Inventory (MAI) | | | | |
| A1 | 0.347 | .33 | .77 | .98 |
| A3 | 0.537 |  |  |  |
| A4 | 0.614 |  |  |  |
| A11 | 0.554 |  |  |  |
| A12 | 0.604 |  |  |  |
| A13 | 0.612 |  |  |  |
| A14 | 0.448 |  |  |  |
| A15 | 0.781 |  |  |  |
| A16 | 0.778 |  |  |  |
| A17 | 0.835 |  |  |  |
| A18 | 0.728 |  |  |  |
| A19 | 0.641 |  |  |  |
| A20 | 0.589 |  |  |  |
| A23 | 0.679 |  |  |  |
| A24 | 0.482 |  |  |  |
| A25 | 0.762 |  |  |  |
| Perceived Maternal Parenting Self-Efficacy Questionnaire (PMPS-E) | | | | |
| B1 | 0.396 | .74 | .84 | .84 |
| B2 | 0.44 |  |  |  |
| B4 | 0.685 |  |  |  |
| B5 | 0.646 |  |  |  |
| B6 | 0.611 |  |  |  |
| B7 | 0.586 |  |  |  |
| B8 | 0.709 |  |  |  |
| B13 | 0.731 |  |  |  |
| B18 | 0.637 |  |  |  |
| B3 | 0.603 | .62 | .82 | .87 |
| B14 | 0.809 |  |  |  |
| B15 | 0.744 |  |  |  |
| B16 | 0.724 |  |  |  |
| B17 | 0.503 |  |  |  |
| B9 | 0.832 | .50 | .90 | .74 |
| B10 | 0.875 |  |  |  |
| B11 | 0.821 |  |  |  |
| B12 | 0.783 |  |  |  |
| Postpartum Depression Screening Scale (PDSS) | | | | |
| C1 | 0.403 | .65 | .96 | .98 |
| C2 | 0.584 |  |  |  |
| C3 | 0.584 |  |  |  |
| C4 | 0.616 |  |  |  |
| C5 | 0.685 |  |  |  |
| C6 | 0.587 |  |  |  |
| C7 | 0.597 |  |  |  |
| C8 | 0.571 |  |  |  |
| C9 | 0.759 |  |  |  |
| C10 | 0.75 |  |  |  |
| C11 | 0.742 |  |  |  |
| C12 | 0.786 |  |  |  |
| C13 | 0.712 |  |  |  |
| C14 | 0.632 |  |  |  |
| C15 | 0.486 |  |  |  |
| C16 | 0.625 |  |  |  |
| C17 | 0.799 |  |  |  |
| C18 | 0.73 |  |  |  |
| C19 | 0.75 |  |  |  |
| C20 | 0.585 |  |  |  |
| C21 | 0.455 |  |  |  |
| C22 | 0.531 |  |  |  |
| C23 | 0.47 |  |  |  |
| C24 | 0.761 |  |  |  |
| C25 | 0.61 |  |  |  |
| C26 | 0.704 |  |  |  |
| C27 | 0.463 |  |  |  |
| C28 | 0.353 |  |  |  |
| C29 | 0.618 |  |  |  |
| C30 | 0.464 |  |  |  |
| C31 | 0.735 |  |  |  |
| C32 | 0.754 |  |  |  |
| C33 | 0.772 |  |  |  |
| C34 | 0.778 |  |  |  |
| C35 | 0.544 |  |  |  |
| AVE = Average variance extracted; CR = Composite reliability | | | | |
